# Near-infrared brain imaging study of depressive disorder with liver qi stagnation syndrome before and after treatment

**DOI:** 10.3389/fpsyt.2025.1529575

**Published:** 2025-03-11

**Authors:** Gui-Fang Chen, Meng-Chai Mao, Chen-Chao Yu, Kun Feng, Xin-Yu Wang, Dong-Sheng Xu, Po-Zi Liu

**Affiliations:** ^1^ Department of Psychiatry, Zunyi Medical University, Affiliated Hospital of Zunyi Medical University, Zunyi, Guizhou, China; ^2^ School of Clinical Medicine, Tsinghua University, Beijing, China; ^3^ School of Rehabilitation Science, Shanghai University of Traditional Chinese Medicine, Shanghai, China; ^4^ Engineering Research Center of Traditional Chinese Medicine Intelligent Rehabilitation, Ministry of Education, Shanghai, China; ^5^ Institute of Rehabilitation Medicine, Shanghai Academy of Traditional Chinese Medicine, Shanghai, China; ^6^ Independent Researcher, Beijing, China; ^7^ Department of Psychiatry, Yuquan Hospital, Tsinghua University, Beijing, China; ^8^ Center of Rehabilitation Medicine, Yueyang Hospital of Integrated Traditional Chinese and Western Medicine, Shanghai University of Traditional Chinese Medicine, Shanghai, China

**Keywords:** depressive disorder, depression, liver qi stagnation, fNIRS, VFT

## Abstract

**Objective:**

This study is a cohort study of depressive disorder patients with liver qi stagnation syndrome specified under the theory of Integrated Chinese and Western Medicine (ICWM). They were measured hemodynamic activity, using fNIRS assessment with verbal fluency task (VFT) pre-treatment and post-treatment, to examine the neurophysiological characteristics, and to explore the impact of drug treatment on it.

**Methods:**

This study recruited patients who were diagnosed with depressive episodes according to the DSM-V diagnostic and Traditional Chinese Medicine (TCM) diagnostic criteria in the outpatient department of Yuquan Hospital Tsinghua University. 35 patients who met the criteria were recruited. All patients were treated with selective serotonin reuptake inhibitors (SSRIs) drugs. The clinical evaluation, VFT and fNIRS assessment were performed pre-treatment and post-treatment two times. After 3 months of treatment, the clinical evaluation, VFT and fNIRS assessment were performed again as a follow-up assessment for the third time.

**Results:**

There were statistically significant differences in HAMD scores between pre-treatment and follow-up assessment (*p* =0.037), with the total scores of HAMD significantly decreased in follow-up assessment. The lDLPFC and mPFC activation in fNIRS during VFT was significantly increased after treatment, as compared to pretreatment assessment, in CH13(*p* = 0.003) and CH30 (*p* = 0.035), and the improvement at lDLPFC remained in the follow-up assessment in CH13 (*p* = 0.007).

**Conclusion:**

This indicates that the activation of the lDLPFC improved after the treatment, and this improvement can remain stable. Hemodynamic activation can reflect the changes of brain function after the one-month treatment, even before the changes of clinical symptoms in depression liver-qi stagnation syndrome. Physiological indicators like fNIRS result may better reflect the improvement of depression liver-qi stagnation syndrome than ethological indicators like HAMD.

## 1 Introduction

Depressive disorder is an affective disorder characterized by low mood, decreased interest, lack of pleasure, and lack of motivation. It is a heterogeneous, complex, and multi-dimensional disorder that is one of the leading causes of disability worldwide (1). The prevalence of depressive disorder is high (2). In 2020, the age-standardized prevalence of depression among adults was 18.5% (3). The prevalence rate of moderate to severe depression among Chinese people was about 8.29% (4). Depressive disorder not only cause emotional problems in patients, but also lead to varying degrees of cognitive impairment.

Since 1950 the medical profession in China raised the theory of Integrated Chinese and Western Medicine (ICWM). ICWM aims to blend the strengths of both medical systems. Traditional Chinese Medicine (TCM) focuses on holistic and natural healing methods, while Western Medicine emphasizes scientific and technological advancements. By integrating these approaches, ICWM seeks to provide more comprehensive and effective healthcare solutions (5). Classic TCM literature recognizes depression under the terms “Qingzhi Disease” with English translation “depression syndrome” or “emotional disease” (6). To accurately diagnose TCM syndromes related to depression, it is necessary to grasp the diagnostic criteria of Western medicine and carry out clinical epidemiological investigations of TCM syndromes with some samples. According to the definition and clinical manifestations of depression in Western medicine, the syndrome differentiation of depression is more concentrated on liver qi stagnation, liver depression transforming into fire, qi stagnation and phlegm obstruction, heart and spleen deficiency, liver and kidney yin deficiency, yin deficiency and fire hyperactivity (7). Among these syndromes, liver qi stagnation syndrome is the most common one observed in clinical. Several research has found that liver qi stagnation accounts for the highest proportion, with one study reporting a prevalence of 27.2% (8) and another 19.1% (9), both indicating that liver qi stagnation has the higher proportion among the various syndromes. it is worth noting that TCM has its unique system and naming in the pathological analysis, treatment, prescription application of depression. Currently, the efficacy evaluation of liver qi stagnation syndrome, particularly in the context of depressive disorder, involves a comprehensive approach that includes both subjective and objective measures. Yet, the clinical approach mostly relies on subjective symptom assessment, including standardized questionnaires and self-reporting by patients, which were susceptible to individual bias. Objective measures involve potential biomarkers using neuroimaging techniques, such as functional magnetic resonance imaging (fMRI) (10) and EEG (11).

Functional Near-Infrared Spectroscopy (fNIRS) is an emerging method that uses infrared light with wavelengths between 650 and 900 nanometers to illuminate the head. It calculates the relative concentration changes of oxygenated and deoxygenated hemoglobin by utilizing hemoglobin’s absorption of near-infrared light. Based on the neurovascular coupling mechanism, it indirectly reflects the regulatory functions of brain. With medium spatial resolution and penetration depth, fNIRS has a relatively high temporal resolution. It has been widely used in various clinical studies, such as depressive disorder, bipolar disorder, schizophrenia, anxiety disorders. One study demonstrates that the fNIRS brain networks show good test-retest (TRT) reliability on resting-state functional connectivity and fair to excellent reliability on most global metrics (12).

Liver qi stagnation syndrome is one of the most common depressive disorder syndromes in TCM, and most depressive disorder patients tend to be associated with cognitive impairment, the higher the degree of depression, the more severe the cognitive impairment, which has a huge impact on the work and life of depressive disorder patients. Therefore, this study measured hemodynamic activity, using fNIRS assessment, in depressive disorder patients during verbal fluency task (VFT) before and after treatment, to examine the neurophysiological characteristics of liver qi stagnation syndrome in the completion of cognitive tasks, and to explore the impact of drug treatment on it.

## 2 Materials and methods

### 2.1 Participants

This study recruited patients who were diagnosed with depressive episodes according to the DSM-V diagnostic criteria in the outpatient department of Yuquan Hospital. 35 patients who met the syndrome of liver qi stagnation were recruited into the cohort study. All patients were treated with selective serotonin reuptake inhibitors (SSRIs) drugs.

The clinical evaluation, VFT, and fNIRS- assessment were performed before and after 1 month of treatment in the liver qi stagnation group. After 3 months of treatment, the clinical evaluation, VFT, and fNIRS assessment were performed again as a follow-up assessment.

The subjects in this study were aged between 18 and 50 years. In addition, subjects with psychiatric symptoms or other psychiatric disorders, chronic substance abuse, serious medical conditions, or intellectual disabilities were excluded. This study was approved by the Ethics Committee of Yuquan Hospital. Clinical registration: ChiCTR2100043338.

### 2.2 Clinical evaluation

Hamilton Depression Scale (HAMD, 24 items) (13), and Hamilton Anxiety Rating Scale (HAMA) (14) were conducted by professional staff to assess depression and anxiety symptoms.

### 2.3 Brain activation task

**Figure 1:**
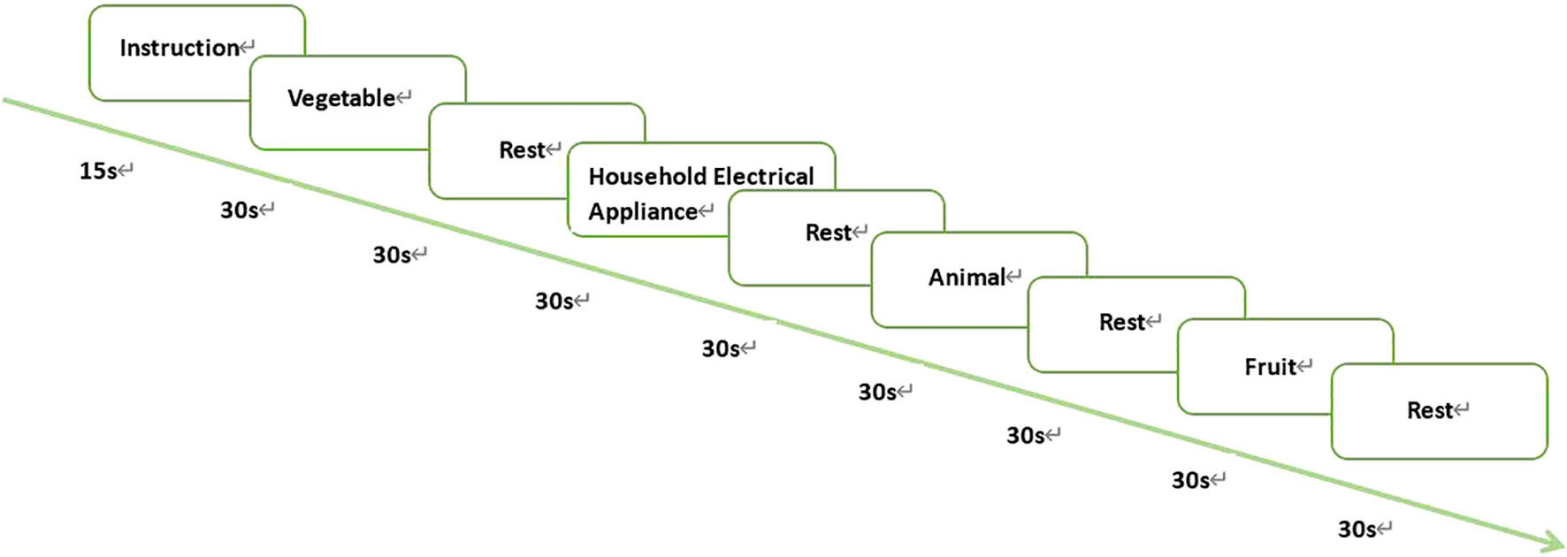
VFT tasks.

The verbal fluency task is a sensitive cognitive task to test depressive disorder and is widely used clinically to evaluate cognitive dysfunction, with a moderate workload on participants (15). Therefore, this task was selected in this study. Participants were asked to say as many words as they could in the given category within 30 seconds, take a 30-second break, and then describe words in another category, with a total duration of four minutes (see **Figure 1**). The order of the four categories is vegetables, household appliances, animals, and fruits. fNIRS measurement began with a 5-minute rest, while participants were asked to sit comfortably before a computer monitor and relax. To reduce motion artifacts in fNIRS data, the participants were asked to minimize physical activity, especially head movement, during VFT and fNIRS measurement. At the end of the experiment, the number of recorded words of each category was used as the score of the VFT tasks.

VFT instructions are visualized on the computer screen by E-Prime2.0.

### 2.4 fNIRS measurement

**Figure 2:**
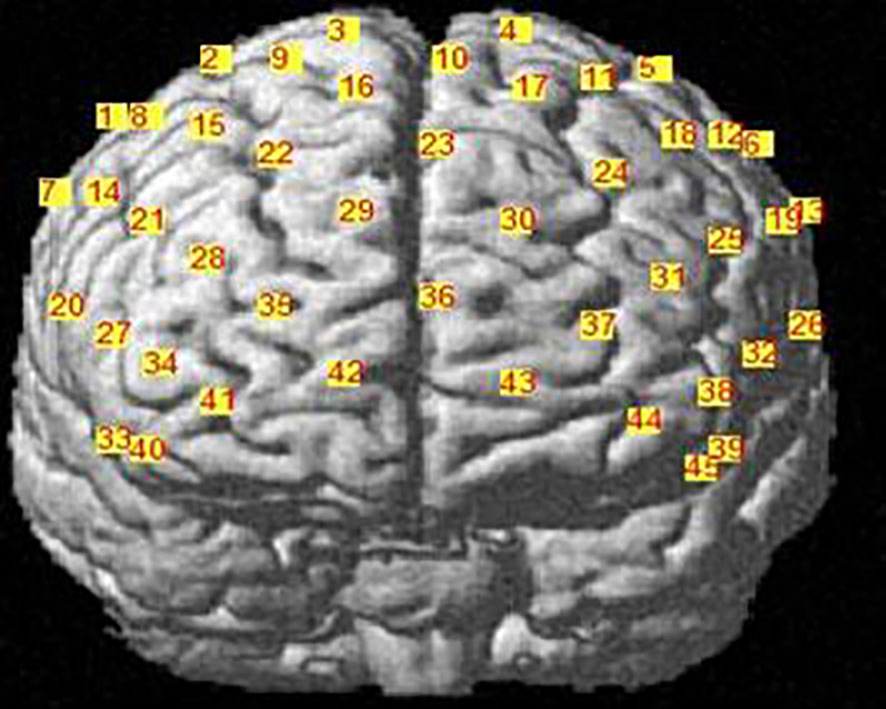
Arrangement of fNIRS channels, frontal view.

**Figure 3:**
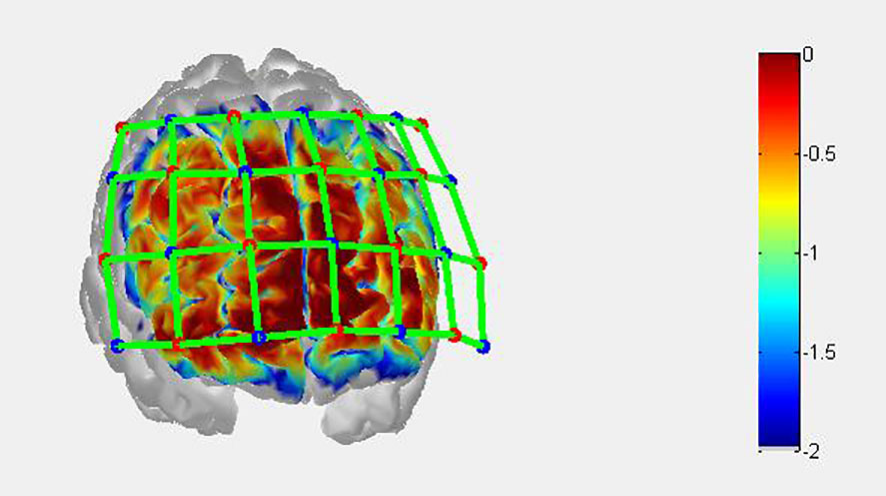
Sensitivity map of fNIRS measurement. measurement sensitivity on the frontal cortex was calculated by Monte Carlo simulation. The red and blue dots represent the light source and detector, respectively, and the green line represents the channel. The color bar represents the spatial sensitivity of fNIRS measurement.

This study uses a 45-channel continuous-wave near-infrared spectroscopy system (FOIRE3000, Shimadzu Corporation, Japan) with three wavelengths (780, 805, and 830 nm). Fourteen emitting and fourteen detecting optrodes were placed on the subjects’ foreheads, with a 3 cm spacing between each pair. Optical optrodes are placed according to the international 10-20 system (16), and the lowest optical optrode is located on the FP1-FP2 line as shown in **Figure 2**. The area between each emitting optrode and detecting optrode, referred to as a channel (CH), corresponds to the cortical region 2-3 cm below the skin and scalp surface (17). The detection area primarily covers the bilateral prefrontal cortex (PFC) (18). In this study, measurement sensitivity in the frontal cortex was calculated using Monte Carlo simulations (19) (see **Figure 3**). Relative changes in hemoglobin were calculated from the absorption of near-infrared light using the modified Beer-Lambert law (20, 21).

### 2.5 Data preprocessing and feature extraction

MATLAB 2013b and NIRs_SPM4.049 were used to preprocess and analyze fNIRS data. To mitigate the influence of motion artifacts on fNIRS data, wavelet transform was applied to the concentration data, and the distribution of wavelet coefficients was calculated. Coefficients exceeding 1.5 times the interquartile range, likely caused by motion artifacts, were set to zero. The fNIRS data also included nonneural signals such as machine noise and physiological noise (e.g., breathing and heartbeat). A Gaussian high-pass filter based on a discrete cosine transform (DCT) algorithm (high-pass filter cut-off: 128s) was used to attenuate those low-frequency noises. A Gaussian low-pass filter (<1 Hz) was used to remove the high-frequency noise.

The General Linear Model (GLM) was used to detect the hemodynamic activation of each participant. The design matrix consisting of the boxcar regressors for VFT was convolved with a Gaussian hemodynamic response function (HRF) to obtain the predictors of hemodynamic activation of each channel. β-value indicating the weight of the task design in the time series of the hemodynamic signal was estimated by the least square principle.

### 2.6 Statistical analysis

SPSS24.0 software package was used for statistical analysis. VFT, clinical rating scale, and the hemodynamic activation are compared by paired t-test to compare the efficacy of treatment at baseline, after 1-month treatment, and after 3-month treatment. For fNIRS data, only oxy-hemoglobin (OxyHb) was analyzed in this study because previous evidence showed that OXY-Hb more accurately reflected brain regional changes than deoxy-hemoglobin (DeoxyHb) (22–24). False Discovery Rate (FDR) correction (FDR corrected p<0.2) was performed for the correction of multiple comparisons. In addition, this study also analyzed the correlation between HAMD/HAMA scores and hemodynamic activation (β-value) of channels with intergroup differences. By comparing the demographic characteristics of the completion group and the dropout group, to make sure that patient dropout is random and does not affect the stability and reliability of the analysis results.

## 3 Results

### 3.1 Clinical and behavioral assessment

The demographic information of patients across study sessions (**Table 1**). There was no significant difference in anxiety, depression, and VFT spoken words between pre-treatment and post-treatment assessment (*p >*0.05). There were statistically significant differences in HAMD scores between pretreatment and the follow-up assessment (*p* =0.037), with the total scores of HAMD significantly decreased in the follow-up assessment. (VFT spoken words and clinical evaluation as shown in **Table 1**).

**Table 1:** Demographic information VFT spoken words and clinical evaluation.

	Pre-treatment (n=35)	Post-treatment (n=35)	Follow-up (n=17)	Post- *vs* Pre-		Follow-up *vs* Pre-	
				*t/χ2*	*p*	*t/χ2*	*p*
Gender	14/21	14/21	6/11	0.000	1.000	0.107	0.744
Age	28.43 ± 9.56	28.83 ± 10.33	27.00± 6.99	-0.168	0.867	0.548	0.586
Education	14.86 ± 2.66	14.66 ± 2.83	15.24 ± 3.03	0.305	0.761	-0.460	0.648
VFT 1	9.63 ± 3.73	10.14 ± 3.61	10.12 ± 2.87	-0.586	0.56	-0.475	0.637
VFT 2	8.00 ± 3.29	8.34 ± 3.08	8.88 ± 3.18	-0.450	0.654	0.917	0.364
VFT 3	9.66 ± 3.70	10.20 ± 3.66	9.82 ± 2.22	-0.617	0.539	-0.171	0.865
VFT 4	9.97 ± 2.77	10.43 ± 3.78	11.06 ± 2.90	-0.577	0.566	-1.307	0.197
HAMA	10.29 ± 6.13	8.23 ± 7.28	7.76 ± 7.00	1.279	0.205	1.328	0.190
HAMD	14.03 ± 8.00	11.80 ± 7.61	8.82 ± 8.58	1.194	0.237	2.149	0.037*

*indicate statistical significance.

### 3.2 Brain activation

#### 3.2.1 The mapping of fNIRS channels on Brodman brain map

**Figure 4:**
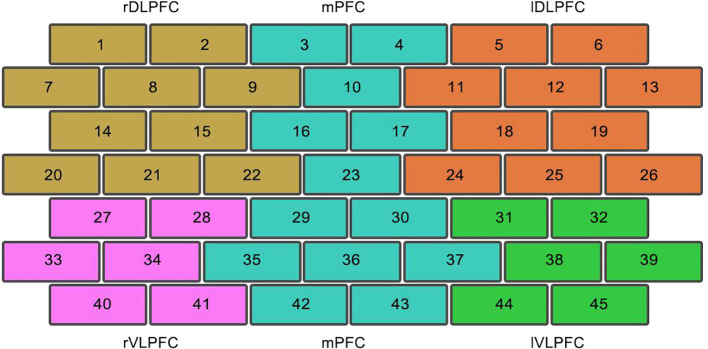
The mapping of 45 fNIRS channels on Brodman brain map. channel 1, 2, 7, 8, 9, 12, 15, 20, 21, and 22 are mapped to rDLPFC, channel 3, 4, 10, 16, 17, 23, 29, 30, 35, 36, and 37 are mapped to mPFC, channel 5, 6,11, 12, 13, 18, 19, 24, 25, and 26 are mapped to lDLPFC, channel 27, 28, 33, 34, 40, and 41 are mapped to rVLPFC, channel 31, 32, 38, 39, 44, and 45 are mapped to lVLPFC.

Acoording to the manual of Shimadzu FOIRE3000, the total 45 fNIRS channels can be mapped to Brodman brain map into 5 areas, which are right dorsolateral prefrontal cortex (rDLPFC), medial prefrontal cortex (mPFC), left dorsolateral prefrontal cortex (lDLPFC), right ventrolateral prefrontal cortex (rVLPFC), and left ventrolateral prefrontal cortex (lVLPFC), as shown in **Figure 4**.

#### 3.2.2 Comparison between pre-treatment and post-treatment assessment

**Figure 5:**
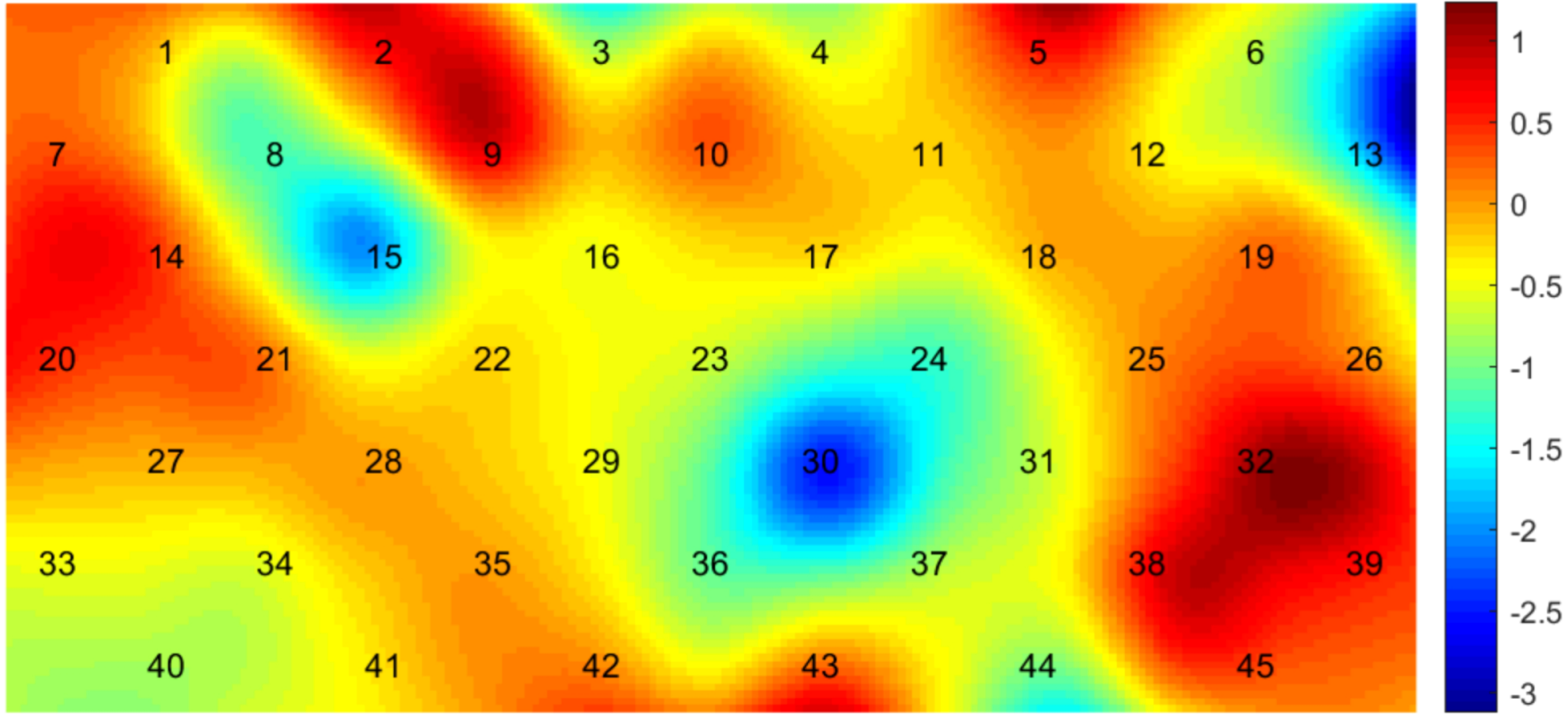
Comparison of hemodynamic activation between pre-treatment and posttreatment assessment. Numbers 1-45 are channels in the figure, which show the activation of the prefrontal cortex during VFT in the liver qi stagnation group between pre-treatment and post-treatment assessment. The bar on the right of the figure shows the range of T values obtained by comparison between the two groups and the corresponding color of T values.

There were statistically significant differences in CH13(lDLPFC, p = 0.003), CH15(rDLPFC, p = 0.039) and CH30 (mPFC, p = 0.035), when comparing the hemodynamic activation between pre-treatment and posttreatment assessment. The hemodynamic activation during verbal fluency tasks significantly increased after treatment, as shown in **Figure 5**.

#### 3.2.3 Comparison of PFC activation between pre-treatment and follow-up assessment

**Figure 6:**
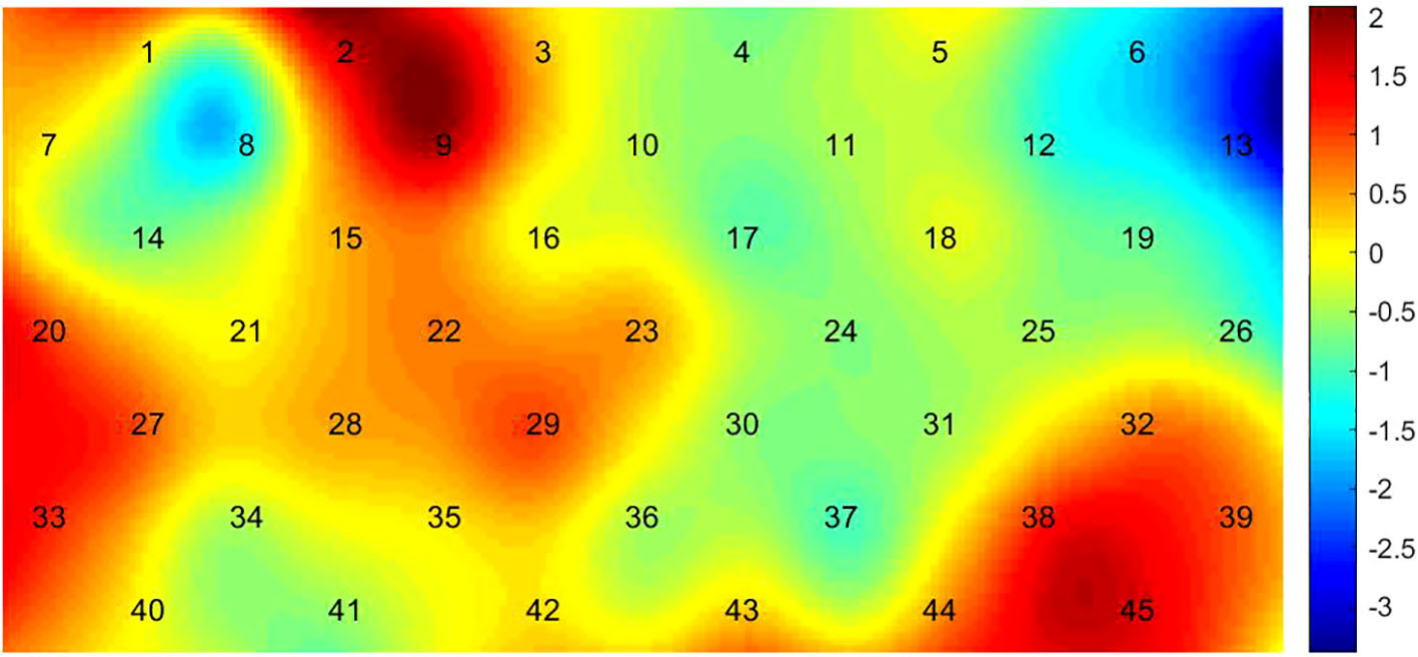
Comparison between pre-treatment and follow-up. Numbers 1-45 are channels in the figure, which show the contrast of hemodynamic activation of patients with liver qi stagnation during the verbal fluency task between pre-treatment assessment and follow-up assessment. The bar on the right of the figure shows the range of T values and the corresponding color of T values compared between the two groups. The larger the absolute value of T, the darker the color, and the greater the difference.

The hemodynamic activation in CH13 in follow-up assessment was significantly (*p* = 0.007) stronger than that in pre-treatment assessment, as shown in **Figure 6**.

#### 3.2.4 Correlation analysis

In this study, the correlation between the total HAMD/HAMA score and positive hemodynamic activation score was calculated, and no significant correlation was observed

#### 3.2.5 Sensitively analysis

The demographic characteristics of the completed follow-up group and the dropout group, such as gender, age, education, handedness, and baseline total scores of the two questionnaires, were analyzed, with all p-values greater than 0.05. This means that the dropout occurred randomly and does not significantly affect the statistical results.

As shown in **Table 2**:

**Table 2:** Sensitivity analysis of Follow-Up and Dropout Groups.

Demographic Characteristics	Follow-Up Group (N=17)^1^	Dropout Group (N=18)	P-value^2^
Gender			0.581
Male	6 (35.29%)	8 (44.44%)	
Female	11 (64.71%)	10 (55.56%)	
Handedness			>0.999
Right-handed	16 (94.12%)	16 (88.89%)	
Left-handed	1 (5.88%)	2 (11.11%)	
Education			0.210
Middle school	2 (11.76%)	1 (5.56%)	
High school	2 (11.76%)	4 (22.22%)	
College Diploma	4 (23.53%)	2 (11.11%)	
Bachelor’s	6 (35.29%)	11 (61.11%)	
Master’s	3 (17.65%)	0 (0.00%)	
Age	26.88 ± 7.01	29.11 ± 9.83	0.643
Baseline HAMA Score	10.76 ± 6.28	9.56 ± 5.89	0.574
Baseline HAMD Score	13.06 ± 6.64	14.44 ± 8.75	0.895

^1^n (%); Mean ± SD.

^2^Pearson’s Chi-squared test; Fisher’s exact test; Wilcoxon rank sum test.

## 4 Discussion

According to the literature survey results, this is the first study to examine the characteristics of fNIRS based on the VFT task before and after treatment of liver qi stagnation syndrome in depressive disorder. There were statistically significant differences in HAMD scores between pre-treatment and the follow-up assessment, with the total scores of HAMD significantly decreased in the follow-up assessment. The fNIRS results showed that the activation of lDLPFC in the liver qi stagnation patients differed between pre-treatment and post-treatment assessment, and the functional improvement sustained after 3 months of treatment.

### 4.1 Clinical and VFT performance of liver qi stagnation group

In terms of the depression and anxiety scores, there was no significant difference between pre-treatment and post-treatment assessment of HAMD, but a slight trend of decrease was observed. The total scores of HAMD after 3 months of treatment were significantly lower than those pre-treatment, indicating that the symptoms of depression improved after 3 months of treatment, which was consistent with previous studies (25). Previous studies showed that antidepressants were effective for 50-70% of depressive disorder patients, but the treatment effect may be delayed. The reason is that it takes a certain time for the neurons to regenerate and increase after the blood concentration of antidepressants is reached, to restore and enhance the information transmission function in neural pathways (26). SSRIs and SNRIs have high safety and usually take effect in 2-4 weeks after medication (27). Therefore, there was no significant difference between the total scores of HAMD in this study at pre-treatment and post-treatment assessment. After 3 months of treatment, the patients’ total scores of HAMD were significantly lower than that before treatment, showing an obvious treatment effect.

There was no significant difference in VFT before treatment, 1 month after treatment and 3 months after treatment. There was no correlation between SSRI efficacy and VFT performance. It is consistent with the results of previous studies. Researches show that SSRI’s frequently do not treat to full remission, and can cause cognitive blunting—actually adding to cognitive problems (28). Therefore, the improvement of cognitive impairment in depression still needs to be further explored.

### 4.2 Comparison of the liver qi stagnation group pre-treatment, post-treatment, and follow-up

The hemodynamic activation of pre-treatment assessment of the lDLPFC and mPFC (CH13 and CH30) was improved after treatment, which is consistent with the changes of brain functional areas in patients with liver qi stagnation syndrome were mainly on the left side. Previous studies have found that the amplitude of P300 of electrode points FZ, FP1, and FP2 in the frontal area of patients with depressive disorder liver depression syndrome is significantly higher than that of pretreatment (29), indicating that treatment can improve brain function. It is consistent with our finding, which means brain function of patients with liver qi stagnation can be improved after drug treatment. The reason is that the hemodynamic activity in the frontotemporal cortex is related to the effect of depressive disorder on SSRI (30). By using fNIRS assessment, enhanced PFC function could be observed after treatment. And the efficacy was maintained until 3 months follow-up in CH13. This indicates that the activation of the lDLPFC improves with the treatment of the disease, and this improvement can remain stable. Previous studies have shown that fNIRS can be used as an auxiliary means to monitor the therapeutic effect of depressive disorder (31). According to the results, it is speculated that the lDLPFC CH13 is a sensitive indicator for monitoring the efficacy of response therapy. This study verified that frontal hemodynamic activity can be used as a potential biomarker to reflect the changes of depressive disorder TCM syndromes before and after treatment.

### 4.3 Limitations

This study only examined the tracking results of the treatment of a small group of depressive disorder patients with liver qi stasis syndrome. In future studies, the sample size will be further expanded, measures will be taken to reduce the dropout of subjects, and the neurophysiological characteristics of TCM syndrome type of depression and the changes in the imaging of disease transmission and outcome of this syndrome type after treatment will be further investigated. At the same time, the neurophysiological characteristics of other TCM syndromes of depressive disorder and the changes in the imaging of disease transmission and outcome are encouraged to be collected to provide empirical evidence for the diagnosis and treatment of TCM syndromes of depressive disorder.

## 5 Conclusion

The hemodynamic activation of lDLPFC in depression patients with liver qi stagnation was improved after 1 month of drug treatment according to the result of fNIRS study, and the therapeutic effect was sustained for 3 months. Even though there was no significant improvement in HAMD score before- and after treatment. This suggests that physiological indicators such as fNIRS results may play an important role in reflecting the improvement of liver qi stagnation syndrome in depression after treatment.

fNIRS has the potential to be used as a sensitive indicator to monitor and detect the therapeutic effects of depressive disorder with liver qi stagnation. This study provides an empirical basis for the diagnosis and therapeutic effect monitoring of TCM syndromes of depressive disorder. Further research is beneficial to the integration of traditional Chinese and Western medicine and the development of Chinese medicine.

## Data Availability

The raw data supporting the conclusions of this article will be made available by the authors, without undue reservation.
